# Antifungal Mechanism of Vip3Aa, a Vegetative Insecticidal Protein, against Pathogenic Fungal Strains

**DOI:** 10.3390/antibiotics10121558

**Published:** 2021-12-20

**Authors:** Seong-Cheol Park, Jin-Young Kim, Jong-Kook Lee, Hye Song Lim, Hyosuk Son, Su-Hyang Yoo, Seong-Eun Mun, Mi-Kyeong Jang, Jung Ro Lee

**Affiliations:** 1 Department of Chemical Engineering, Sunchon National University, Suncheon 57922, Korea; schpark9@gnu.ac.kr (S.-C.P.); jyfrog@hanmail.net (J.-Y.K.); seal9669@naver.com (J.-K.L.); skitten74@mabik.re.kr (H.S.); 2 LMO Research Team, National Institute of Ecology, 1210 Geumgang-ro, Maseo-myeon, Seocheon-gun 33657, Korea; hslim0826@nie.re.kr (H.S.L.); hyang77@nie.re.kr (S.-H.Y.); 3 National Marine Biodiversity Institute of Korea, 101-75 Jangsan-ro, Janghang-eup, Seocheon-gun 33662, Korea; 4 Department of Biological Science, College of Natural Science, Wonkwang University, Iksan 54538, Korea; showmse@nate.com

**Keywords:** antifungal activity, Vip3Aa protein, reactive oxygen species, *Bacillus thuringiensis*

## Abstract

Discovering new antifungal agents is difficult, since, unlike bacteria, mammalian and fungal cells are both eukaryotes. An efficient strategy is to consider new antimicrobial proteins that have variety of action mechanisms. In this study, a cDNA encoding *Bacillus thuringiensis* Vip3Aa protein, a vegetative insecticidal protein, was obtained at the vegetative growth stage; its antifungal activity and mechanism were evaluated using a bacterially expressed recombinant Vip3Aa protein. The Vip3Aa protein demonstrated various concentration- and time-dependent antifungal activities, with inhibitory concentrations against yeast and filamentous fungi ranging from 62.5 to 125 µg/mL and 250 to 500 µg/mL, respectively. The uptake of propidium iodide and cellular distributions of rhodamine-labeled Vip3Aa into fungal cells indicate that its growth inhibition mechanism involves its penetration within cells and subsequent intracellular damage. Furthermore, we discovered that the death of *Candida albicans* cells was caused by the induction of apoptosis via the generation of mitochondrial reactive oxygen species and binding to nucleic acids. The presence of significantly enlarged Vip3Aa-treated fungal cells indicates that this protein causes intracellular damage. Our findings suggest that Vip3Aa protein has potential applications in the development of natural antimicrobial agents.

## 1. Introduction

*Bacillus thuringiensis* is the most widely used biological insecticide for controlling insect pests, primarily Lepidoptera and Coleoptera species [1]. Among the insecticidal proteins secreted by *B. thuringiensis*, the parasporal inclusion crystal (Cry) toxins are the most well-known and widely used. Cry toxins accumulate during sporulation in the *B. thuringiensis* strain, resulting in a crystalline inclusion with a variety of morphologies. When pests consume Cry toxins, the alkaline digestive tract of the insects denatures the insoluble crystals, making them soluble and thus susceptible to digestion by proteases found in the pest gut, which releases the toxin from the crystal [2]. Following this, the Cry toxin penetrates the cell membrane of the pest digestive tract, paralyzing the gut and forming a pore. The pest eventually stops eating and starves to death [2,3].

Vegetative insecticidal protein (Vip) is another type of insecticidal protein produced by *B. thuringiensis* and *B. cereus* [3,4,5,6,7]. Vip proteins are released during vegetative growth and have no resemblance to Cry toxins. Until recently, the Vip protein family was divided into four categories: Vip1, Vip2, Vip3, and Vip4 [8]. Vip1 and Vip2 proteins are the two parts of a binary toxin, which is toxic to coleopterans. Vip1Aa1 and Vip2Aa1 have high anti-corn rootworm activity [6,9]. The currently known insecticidal molecular mechanism of Vip1Ac toxins begins with the larva ingesting the toxin. The monomer of Vip1Ac activated by the protease in the larva’s midgut forms oligomers containing seven Vip1 molecules [10]. These oligomers bind to specific receptors on the mid-gut border membrane, where the Vip1Ac toxin is then inserted.

Vip3 proteins have a diverse host range, which includes a number of major lepidopteran pests [3,4,5,6,7,8,9]. Vip3A proteins must be cleaved by proteases prior to the recognition of specific 80 kDa and 100 kDa membrane proteins, different from those perceived by Cry toxins at the surface of the mid-gut epithelium [11]. Among the Vip3 family, Warren’s study identified Vip3Aa (89 kDa), which has high toxicity to *Agrotis ipsilon* and other lepidopteran larvae [6]. Vip3A protein has recently been discovered to be a pore-forming protein capable of forming stable ion channels in the membrane [11]. The pH of the solution is one of the factors that may influence the insecticidal activity of the Vip3Aa protein. The pH of lepidopteran midgut lumen ranges from 8.0 to 12.0 [12,13]. Proteolysis by mid-gut proteases is most effective at pH 10.0–12.0. An alkaline condition is required for the Vip3Aa toxin to be converted to its toxic form [12]. However, the effect of pH on the functional properties of Vip3Aa is yet to be determined.

The positively charged hydrophobic region at the N-terminus of Vip3 proteins suggests that this region is important for protein structure and insecticidal activity [4,14,15,16,17]. Furthermore, the last amino acid at the C-terminus is known to play a role in Vip3 protein activity and safety. Most Vip3 proteins have a carbohydrate binding motif, which spans from position 536 to a position near amino acid 652 at the C-terminus [14,15]. Furthermore, recent research has shown that both 19–22 kDa (N-terminus region) and 62–66 kDa (C-terminus region) fragments are required for the stability and specificity of Vip3A toxins [18]. A 340 kDa homo-tetramer composed of the 19–22 kDa and 62–66 kDa fragments of Vip3A was identified after digestion with trypsin or insect mid-gut proteases [19,20]. Quan Y et al., discovered that Vip3Af mutants form structurally diverse oligomers, and Ensi Shao et al. discovered that protein oligomers formed of 19 kDa and 65 kDa fragments of Vip3Aa were critical for insecticidal toxicity [21,22].

While studying the insecticidal activity of the Vip3Aa protein, we developed an interest in the characteristics and novel functions of the Vip3Aa protein, which are similar to those of common antimicrobial peptides (AMPs) [22]. In most organisms, AMPs are involved in the innate host defense system as a primary barrier against infection. Research on AMPs has focused on their abundance in nature, their mechanisms, and their roles in immune systems. The aim of this study was to confirm the pH-dependent antimicrobial activity potential of Vip3Aa protein. As a result, the Vip3A protein demonstrated antifungal activity only at a specific pH. In addition to its insecticidal activity, use of Vip3Aa as a potential antibiotic as part of a biological control strategy was also confirmed.

## 2. Results

### 2.1. Antifungal Activity of Vip3Aa Protein against Pathogenic Fungi

**Figure 1:**
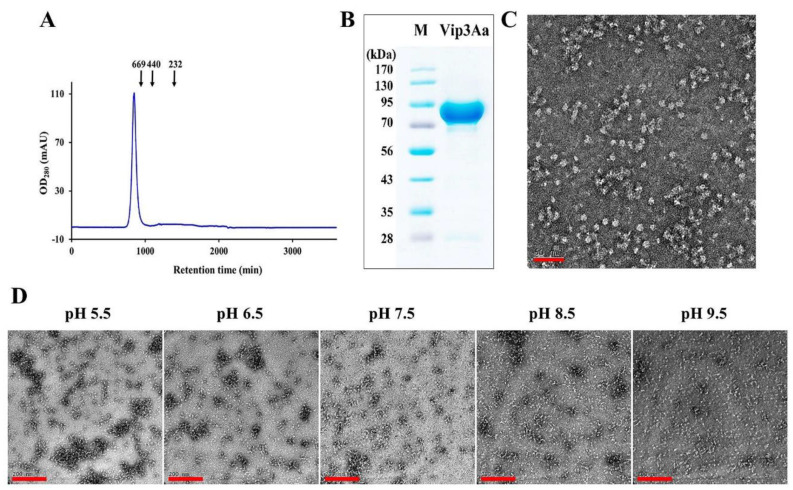
Purification and characterization of recombinant Vip3Aa protein. (**A**) Recombinant Vip3Aa protein was purified on SEC. The short vertical lines indicate standard markers that were calibrated with bovine thyroglobulin (669 kDa), ferritin (440 kDa), and bovine catalase (232 kDa). (**B**) The purified recombinant Vip3Aa from E. coli was resolved by 10% SDS-PAGE. (**C**) Oligomeric forms of Vip3Aa protein fractionated from SEC (pH 9.5) were observed under TEM. Bar presents 50 nm. (**D**) TEM analysis of self-aggregation of Vip3Aa protein in a pH-dependent manner. Bars present 200 nm.

To determine the antimicrobial activity of B. thuringiensis Vip3Aa, we expressed recombinant Vip3Aa protein in *Escherichia coli*. The full-length cDNA of Vip3Aa was cloned into the pET28a vector and expressed in *E. coli* BL21(DE3). To confirm the structural orientation and purity of the isolated recombinant protein, size-exclusion chromatography (SEC) and 10% sodium dodecyl sulphate-polyacrylamide gel electrophoresis (SDS-PAGE) analyses were performed (Figure 1A,B). Analysis of SEC purified fractions revealed that the native molecular weight of Vip3Aa was 669 kDa, which may have an oligomeric structure of at least an octamer or more or an irregular highly aggregated structure (Figure 1A). It is known that the C-terminus of Vip3A proteins are cleaved by midgut proteases to produce a 62–66 kDa protease-resistant toxic core [8,23]. Recently, an approximately 340 kDa homo-tetramer structure has been identified from Vip3A digested by trypsin or insect midgut proteases [19,21,22,24]. However, the native structure of Pro-Vip3Aa has not been clearly identified. Negative staining on a transmission electron microscope (TEM) revealed that the forms of Vip3Aa proteins fractionated on SEC represent a regular oligomer (Figure 1C). These physicochemical analyses show that recombinant Vip3Aa was obtained in a purified form, and this protein has oligomeric structures in its native state. In addition, pH is a very important factor in the insecticidal function of Vip3Aa. Therefore, the pH-dependent structural changes of Vip3Aa protein were confirmed using TEM. In assays from pH 5.5 to 9.5, the proteins of Vip3Aa formed significant self-aggregated complexes, which increased in size by acidification (Figure 1D). We visually confirmed abnormal precipitates in the protein solution under weakly acidic conditions. This may be the initial state of protein crystallization, but it is an important factor in the antifungal activity of the Vip3Aa protein proposed in this study.

In this study, the antifungal activity of Vip3Aa was investigated by determining the inhibitory concentration (IC) against seven fungi species. Melittin, which is derived from honeybee venom, is well known as a natural peptide with high cytotoxicity as well as excellent antimicrobial activity via membranolytic action [25,26]. Because it exists as an ordered self-aggregate in aqueous solution and forms toroidal pores in fungal cells [25,26], we used it as a comparative control. Melittin inhibited the growth of tested fungal cells at IC_50_ values ranged from 11 to 88 µM, while IC_50_ values of Vip3Aa ranged from 0.7 to 5.6 µM (Table 1). The MIC values determined after 24 h or 48 h treatments of Vip3Aa were 1.4 to 2.8 µM against yeast cells and 5.6 to 11.1 µM against mold cells. The molecular weight of the monomeric melittin is about 2.8 kDa, and the recombinant Vip3Aa is about 90 kDa, which is a 32-fold difference. Furthermore, melittin exists as a tetramer and Vip3Aa forms an oligomer as at least an octamer in aqueous solution. When compared with molar concentration, it indicates that the antifungal activity of Vip3Aa is more potent than that of melittin. The antifungal activity of both proteins was better against yeast cells than mold cells within the tested fungal cells.

**Figure 2:**
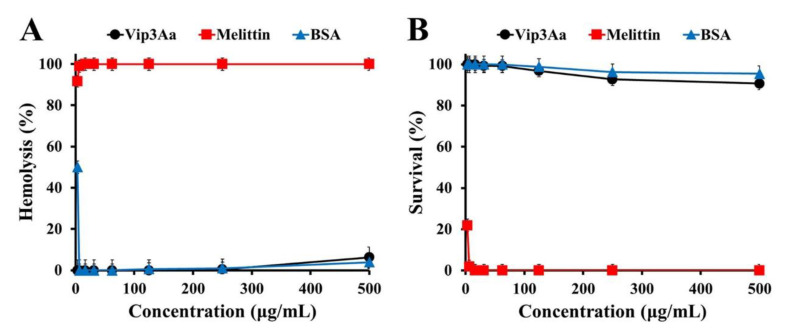
Hemolysis and cytotoxicity of Vip3Aa against rat erythrocytes (**A**) and HaCaT cells (**B**). (**A**) After 1 h incubation in the presence of proteins at the represented concentration, the absorbance of hemoglobin released from rat erythrocytes was measured at 414 nm. (**B**) After 24 h incubation of proteins in HaCaT cells, cell survival was assayed by activated-XTT solution.

To be used as an antifungal agent, all compounds or materials have to possess non-toxic character. We examined the hemolytic and cytotoxic effects on rat erythrocytes and human HaCaT cells, respectively. As shown in Figure 2A, melittin exhibited 91.7% hemolysis even at a low concentration of 3.125 µg/mL, whereas Vip3Aa and BSA achieved 6.3% and 3.9% hemolysis, respectively, at a high concentration of 500 µg/mL. On the other hand, in HaCaT cells, human keratinocytes, melittin at 6.25 µg/mL resulted in 1.9% cell survival, but the cells treated with 500 µg/mL of Vip3Aa and BSA survived to 90.7% and 95.4%, respectively (Figure 2B). Although Vip3Aa causes extensive damage in the midgut of insects (a eukaryote) by forming transmembrane pores, these findings suggest that it causes cell-selective death at least in fungal and human cells. Recently, Yang et al., reported that Cry protein, an insecticidal protein that is similar to Vip3Aa, can be safely used as a peptide delivery carrier in vivo [27].

### 2.2. Molecular Mechanism of the Vip3Aa Protein in Fungal Cells

**Figure 3:**
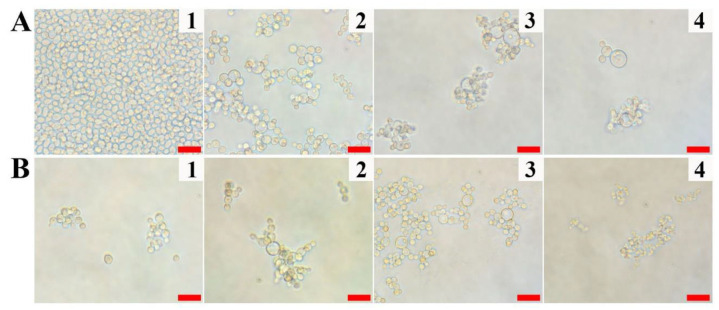
Concentration and time-dependent antifungal effects of recombinant Vip3Aa in *C. albicans* cells. (**A**) Concentration-dependent inhibition of fungal growth at 12 h incubation. (**1**): Control, (**2**): 31.3 µg/mL Vip3Aa, (**3**): 62.5 µg/mL Vip3Aa, (**4**): 125 µg/mL Vip3Aa. (**B**) Time-dependent inhibition of fungal growth by 62.5 µg/mL Vip3Aa. (**1**): 4 h, (**2**): 12 h, (**3**): 24 h, (**4**): 48 h. Bars represent 20 µm.

As shown in Table 1, Vip3Aa was found to possess antifungal properties against yeast and filamentous fungi. Before the mechanism study, the visible growth patterns or morphological changes of *C. albicans* cells according to the concentration and incubation time of Vip3Aa were observed under a microscope (Figure 3A,B). As shown in Figure 3A, depending on the treated Vip3Aa concentration, cell proliferation was significantly inhibited, compared to the control. The morphologies of Vip3Aa-treated *C. albicans* cells were ring-shaped (Figure 3A,B(2–4)), despite the fact that their normal phenotypes are oval (Figure 3A,B(1)). Since the number and size of ring-shaped cells vary upon the treating Vip3Aa concentration, the antifungal effects and cell morphologies of recombinant Vip3Aa were studied in *C. albicans* cells for a 4–48 h incubation period. As a result, the size of ring-shaped cells increased for 24 h, but most of the cells shrank, and cell debris increased after 48 h. This can occur by inhibiting cell wall biosynthesis or cell division or by inducing apoptosis (Figure 3B). Yeast cells generally produce daughter cells by budding and continued cell proliferation. Under normal conditions, chain-type budding occurs rarely. However, chain-type budding was observed in most of the Vip3Aa-treated *C. albicans* cells at low concentration or for short incubation time. Vip3Aa may act on *C. albicans* cells via intracellular damage rather than causing direct membrane damage.

We propose that the antifungal activity of the protein may be due to its structural alternation from a non-aggregated quaternary structure to the aggregated form via changes in intracellular pH environment values of fungal cells.

### 2.3. Intracellular Localization and Molecular Mechanism of Vip3Aa in Fungal Cells

**Figure 4:**
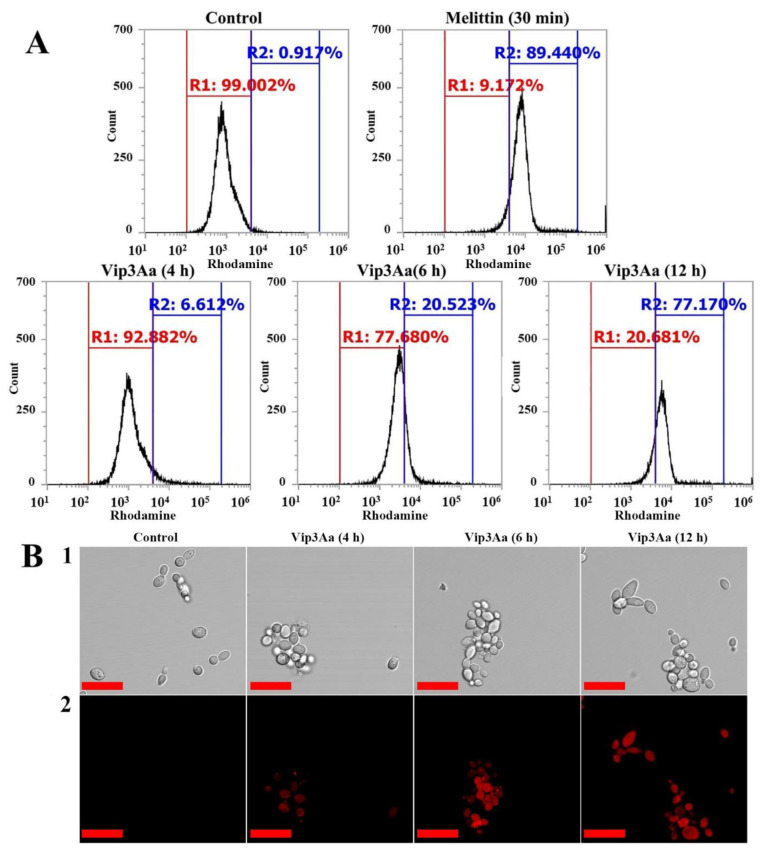
Cellular binding and uptake of Vip3Aa in C. albicans cells. Rhodamine-labeled melittin and Vip3Aa were incubated for the given time points at the IC_50_ concentration, and the treated cells were analyzed using (**A**) flow cytometry and (**B**) confocal laser scanning microscope (CLSM). Bar is 20 µm. 1: DIC, 2: Rhodamine.

To investigate the cell affinity of Vip3Aa, rhodamine was conjugated to amine group proteins. Due to their strong hydrophobicity, fluorescent dyes participate in unwanted side reactions by completely different mechanisms when treated with cells. Hence, we mixed rhodamine-labeled protein and rhodamine-free protein in a weight ratio of 1:9, and *C. albicans* cells were treated with the mixtures at IC_50_. As shown in Figure 4A, cells with melittin showed a fluorescence shift of 89.45% after 30 min of treatment, but Vip3Aa induced a shift of 6.61, 20.52, and 77.18% after 4, 6, and 12 h, respectively. The binding affinity of proteins to cells and their uptake capacity into cells cannot be determined by flow cytometry alone. Therefore, the cellular distribution of Vip3Aa was visualized using a confocal laser scanning microscope (CLSM) (Figure 4B). Cellular red fluorescence was strong after 6 h incubation, but the intracellular accumulation of rhodamine-labeled Vip3Aa increased in all treated cells after 12 h of incubation.

**Figure 5:**
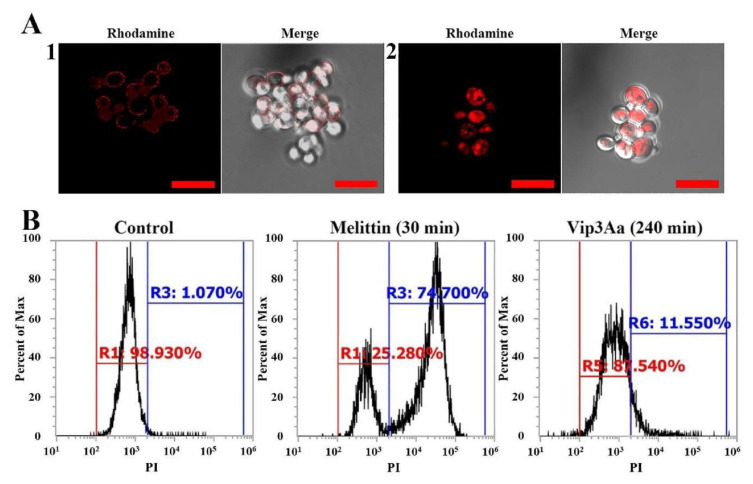
Cellular distributions of Vip3Aa in *C. albicans* cells. (**A**) After incubating *C. albicans* cells with rhodamine-labeled melittin (**1**) for 1 h and Vip3Aa (**2**) for 12 h, the fungal cells were washed and examined using a CSLM. Bar is 10 µm. (**B**) Flow cytometry analysis was used to evaluate intracellular uptake of PI in *C. albicans* cells after incubation with melittin or Vip3Aa.

To determine the cellular localization of proteins in fungal cells, *C. albicans* cells were treated with rhodamine-labeled melittin and Vip3Aa and observed using a CLSM. On the surface of *C. albicans*, the fluorescence of rhodamine-labeled melittin was observed. In contrast, rhodamine-labeled Vip3Aa fluoresced in the cytosol of *C. albicans* (Figure 5A). These findings imply that Vip3Aa has a different mechanism of action from melittin. A propidium iodide (PI) uptake assay was performed using a flow cytometer to confirm the results obtained (Figure 5B). Because PI is an impermeable dye, it can be penetrated into only cells with membrane damage, and it emits red fluorescence by binding to nucleic acids. Melittin is well known for disrupting fungal membranes by forming pores. Thus, melittin exhibited a rapid PI uptake of 74.7% after incubating for 30 min. However, Vip3Aa-treated cells did not show significant PI uptake even after incubation for 240 min. Thus, we propose that the Vip3Aa protein has potent antifungal activity without membranolysis.

### 2.4. Intracellular ROS Production and Apoptosis Induction by Vip3Aa

**Figure 6:**
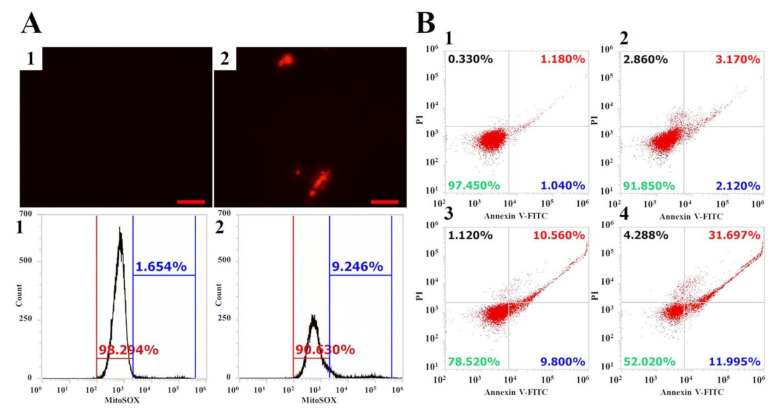
Generation of mitochondrial superoxide (MitoSOX) and induction of apoptosis by Vip3Aa. (**A**) After incubation of Vip3Aa, MitoSOX probe was added in the cells and analyzed by fluorescence microscopy and flow cytometry. (**1**): control, (**2**): Vip3Aa (62.5 µg/mL). Bar is 50 µm. (**B**) Vip3Aa proteins were incubated with C. albicans cells for 12 h, and the cells were stained with PI/FITC Annexin-V and analyzed using flow cytometry. (**1**): Control, (**2**): 31.3 µg/mL of Vip3Aa, (**3**): 62.5 µg/mL of Vip3Aa, (**4**): 125 µg/mL of Vip3Aa.

The mechanisms of action of numerous AMPs are broadly classified into the following two categories: membranolytic actions such as membrane potential changes and the formation of pores in the cell membrane; and intracellular inhibiting actions such as interfering gene expression, inhibiting enzyme activity, generating reactive oxygen species (ROS), and inducing osmotic pressure [28,29]. There is growing evidence of AMPs inducing cell death by stimulating the production of ROS [30,31,32,33,34]. Although fungal cells repeat the cycle of generating and eliminating intracellular ROS during metabolic pathways, the high levels of ROS damage intracellular lipids, proteins, DNA, organelles, and cell walls [35,36]. To investigate the effects of Vip3Aa on mitochondrial ROS production in *C. albicans*, mitochondrial superoxide (MitoSOX) Red, a selective mitochondrial fluorescence probe, was monitored using fluorescence microscopy and flow cytometry (Figure 6A). In flow cytometry analysis, cells treated with Vip3Aa for 12 h showed a 7.6% increase in MitoSOX production compared to the control, but there are many cells emitting red fluorescence under a fluorescence microscope. This difference is due to excessive cell aggregation and enlarged cell size by the incubation time of 12 h, as shown in cell morphological alterations in Figure 3. To determine whether apoptosis is involved in cell death, the *C. albicans* cells treated with Vip3Aa were observed using FITC Annexin-V (specifically binding to externalized phospatidylserine) and PI (cell membrane integrity) co-staining method (Figure 6B). Cells treated with Vip3Aa showed early apoptosis (2–12%), late apoptosis (3–32%), and necrosis (2–4%). With an increase in the concentration of Vip3Aa, apoptosis significantly increased instead of necrosis. This implies that Vip3Aa not only prevents cell proliferation of *C. albicans* but is also involved in cell death.

### 2.5. Morphological Alterations Caused by Vip3Aa in Fungal Cells

**Figure 7:**
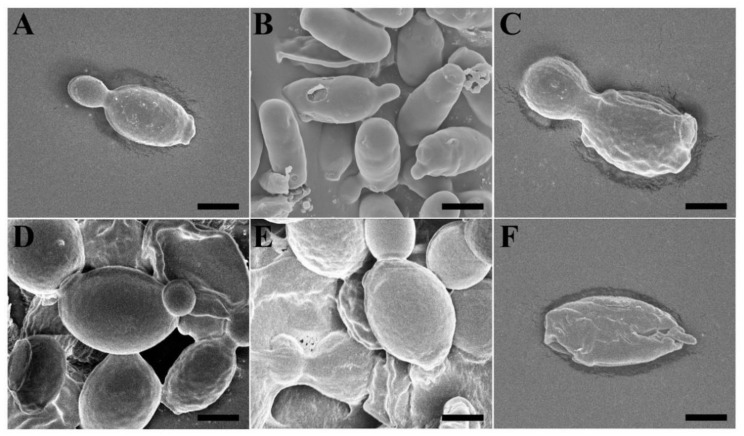
Time-dependent morphological changes in Vip3Aa-treated *C. albicans* cells. (**A**): control, (**B**): melittin (31.3 μg/mL, 4 h incubation), (**C**): Vip3Aa (62.5 μg/mL, 4 h), (**D**): Vip3Aa (62.5 μg/mL, 12 h), (**E**): Vip3Aa (62.5 μg/mL, 24 h), and (**F**): Vip3Aa (62.5 μg/mL, 48 h). Bars represent 1 µm.

Scanning electron microscope (SEM) was used to examine the morphological changes in *C. albicans* cells in the presence of melittin and Vip3Aa. When compared to untreated cells, melittin-treated cells were wrinkled and had irregular-sized holes in the cell surface after 4 h of incubation (Figure 7A,B). Cells treated with Vip3Aa at the IC_50_ level exhibited severely wrinkled cell surfaces and enlarged cell size (Figure 7C–F). The sizes of cells incubated with the Vip3Aa protein were larger than those of control cells, indicating that swelling in the presence of the Vip3Aa protein increased cell size via formation of a single zygote by fusion of yeast cells. The cells incubated with Vip3Aa for 24 h showed a cracked cell wall with surface roughness and numerous scars.

*C. albicans* undergoes hyphal growth when it causes pathogenesis, but it continuously produces daughter cells by budding under normal growth conditions. The most important factor in the antifungal mechanism of Vip3Aa is its structural variations with change in environmental pH. During the growth of *C. albicans* cells, the intracellular pH varies between 6 and 7, owing to glucose metabolism. Penetrated Vip3Aa can be aggregated in acidic cytosol. These can eventually interfere with factors involved in cell division (formation of short cell chains) and induce mitochondrial ROS (cell swelling). In the early stage of Vip3Aa treatment, it inhibits the growth of fungal cells via fungistatic action; however, with time, it develops a fungicidal action that causes cell death via apoptosis.

The main mechanism of antimicrobial peptides is to disrupt the cell membrane via pore formation and permeabilization, leading to rapid death. Because these mechanisms are different from those of conventional antibiotics, many researchers are interested in them as a next-generation antibiotic for antibiotic-resistant bacteria. Most antimicrobial proteins that have relatively large molecular weights penetrate into cells and exhibit antimicrobial actions via ROS generation and inhibition of cell metabolism. However, as shown in the antifungal action of Vip3Aa, it is extremely rare that the cells become ring-shaped and enlarged in size in cell phenotype. Although we suggest mitochondrial ROS generation and intracellular protein aggregation as an antifungal action of VIP3Aa in this study, further studies are needed to define multimodal actions. Studying the antimicrobial action of a protein with a large molecular weight can elucidate the intrinsic function of the protein in each organism, and it is also able to search for a new concept of antibiotic by studying its mechanism. In addition, if we find a domain or motif with antimicrobial effects in the protein sequence, an effective antimicrobial peptide can be designed.

## 3. Materials and Methods

### 3.1. Materials

Annexin V-FITC (Cat. No. A13199), MitoSOX Red (Cat. No. M36008), PI (CAS No. 25535-16-4), 5/6-carboxy-tetramethyl-rhodamine succinimidyl ester (NHS-rhodamine, CAS No. 246256-50-8) were obtained from Thermo Scientific (Waltham, MA, USA). All other reagents were of analytical grade.

### 3.2. Fungal Cells

The fungal cultures were sourced from the Korea Collection for Type Cultures (KCTC, Jeongup-si, Jeollabuk-do, Korea) and Culture Collection of Antimicrobial Resistant Microbes (CCARM, Seoul Women’s University, Seoul, Korea). C. albicans (KCTC 7270), C. krusei (CCARM 14017), C. parapsilosis (CCARM 14016), C. tropicalis (KCTC 7221), Colletotrichum gloeosporioides (KCTC 6169), Fusarium graminearum (KCTC 16656), and F. solani (KCTC 6326) were obtained from KCTC or CCARM.

### 3.3. Cloning and Protein Expression of the Vip3Aa Gene in E. coli

Polymerase chain reaction was used to amplify the Vip3Aa gene from a *B. thuringiensis* cDNA library. The recombinant protein Vip3Aa was expressed in *E. coli* strain BL21(DE3) after being cloned into the pET28a vector. The transformants in BL21 (DE3) cells were cultured at 37 °C in LB medium, and the protein was induced by the addition of 0.4 mM isopropyl-ß-D-thiogalacto-pyranoside (IPTG) for 4 h. The His-tagged Vip3Aa protein was purified on a Ni-NTA affinity column and fast protein liquid chromatography (FPLC) using a Superdex^®^ 200 Increase 10/300 GL column (GE healthcare, Waltham, MA, USA). SDS-PAGE analysis was used to identify intact Vip3Aa protein.

### 3.4. Purification and Structural Analysis of the Vip3Aa Protein

An FPLC (BioLogic DuoFlow Medium-Pressure Chromatography Systems, Bio-Rad, Hercules, CA, USA) system was used to further purify the isolated recombinant Vip3Aa. SEC was performed using a Superdex 200 column on the FPLC system at a flow rate of 0.6 mL/min, using 50 mM sodium borate buffer at 25 °C (pH 9.5). A 10% SDS-PAGE was used to determine the purity of the Vip3Aa protein. Isolated proteins were digested with trypsin and subjected to peptide mass fingerprint analysis using matrix-assisted laser desorption ionization-time of flight (Microflex LRF 20, Bruker Daltonics, Billerica, MA, USA) to identify proteins resolved on SDS-PAGE gel. MASCOT software (available online: http://matrixscience.com; accessed on 15 December 2021) was used to identify the proteins. A transmission electron microscope was used to examine the morphological changes in the Vip3Aa protein purified on SEC. Fractionated proteins were applied to carbon-coated copper grids that had been glow-discharged into the air (Harrick Plasma, Ithaca, NY, USA) and were then negatively stained with 2% uranyl acetate. The grids were examined using a 200 kV FEI Tecnai 20 TEM. Images were captured with a Gatan CCD camera.

### 3.5. Antifungal Assay

Spores of mold fungi grown on potato dextrose (PD; Difco, Sparks, MD, USA) agar plates were collected with PD broth consisting of 0.08% Triton X-100. Yeast cells were subcultured overnight in yeast extract–peptone–dextrose (YPD; Difco) medium. Fungal cells were adjusted to 2 × 10^4^ spores (cells)/mL in phosphate-buffered saline (PBS) (pH 7.4), containing 20% culture medium added to two-fold serially diluted proteins in 96-well plates. After 24 h (for yeast) or 48 h (for mold) incubation at 28 °C, cell growth was examined microscopically with an inverted light microscope. The inhibitory concentration 50 (IC_50_) against each fungus was defined as the lowest concentration of a sample that inhibited 50% visible growth. All assays were performed in triplicate [30,31,32].

### 3.6. Cytotoxicity Assay

Fresh rat blood collected into a sodium heparin-coated tube (BD Vacutainer, Oxford, UK) was centrifuged at 800× *g* for 10 min and washed in PBS until the supernatant was clear. Eight % (*v*/*v*, final concentration) of erythrocytes were added in the serially diluted proteins with PBS. After 1 h incubation at 37 °C with mild agitation, the samples were centrifuged at 800× *g* for 10 min, followed by an absorbance measurement of the supernatant at 414 nm. Each assay was performed in triplicate, and the hemolysis percentage was calculated using the following equation:

% hemolysis = [(Abs_414_ in the protein solution-Abs_414_ in PBS)/(Abs_414_ in 0.1% Triton-X100-Abs_414_ in PBS)] × 100 (1)

100% hemolysis was obtained by the absorbance of 0.1% triton X-100 treatment, and 0% hemolysis was consisted of rat erythrocytes alone in PBS.

In vitro cytotoxicity assay was performed using the 2,3-Bis-(2-Methoxy-4-Nitro-5-Sulfophenyl)-2H-Tetrazolium-5-Carboxanilide (XTT) assay in HaCaT (human keratinocyte) cells. The cells were cultured in Dulbecco’s modified Eagle medium (DMEM; ThermoFisher Scientific, Gibco, Waltman, MA, USA) supplemented with antibiotic-antimycotic (ThermoFisher Scientific, Gibco) and 10% fetal bovine serum (ThermoFisher Scientific, Gibco) at 37 °C in a humidified chamber in an atmosphere containing 5% CO_2_. The cells were seeded at 5 × 10^4^ cells/mL in a 96-well microtiter plate. After 24 h of incubation, the cells were treated with two-fold serial dilutions of proteins, followed by another 24 h of incubation. The plate added by an activated XTT solution was additionally incubated for 4 h, and the absorbance of each well was measured at wavelengths of 480 and 650 nm using a microtiter SpectraMax M5 reader (Molecular Devices, Sunnyvale, CA, USA).

### 3.7. Membrane Integrity Assay Using PI

C. albicans cells were incubated with the indicated proteins at IC_50_, and cells were washed twice with PBS and stained with PI (10 μg/mL). After additional incubation at 4 °C for 30 min, cells were washed twice with PBS to remove unbound dye, and a quantitative analysis was performed using an Attune NxT acoustic focusing cytometer (Thermo Fisher Scientific Co., Waltham, MA, USA).

### 3.8. Analysis Using CLSM

To observe the cellular distribution of proteins, fungal cells were analyzed using a CLSM. After incubation with rhodamine-labeled peptides at 28 °C for the presented times, the washed C. albicans cells were spotted on a cover glass with the mounting solution (50% glycerol, 0.1% n-propyl-gallate) and observed using CLSM (Nikon A1, Nikon Instruments Inc., Tokyo, Japan).

### 3.9. MitoSOX Levels

After incubation of fungal cells treated with proteins at IC_50_ for 12 h, 5 µM of MitoSOX Red probe in PBS was applied to cells, followed by incubation for 10 min at 28 °C in dark. The incubated cells were washed three times with PBS and were analyzed using a fluorescence microscope and flow cytometry (Ex. 530 nm/Em. 575 nm).

### 3.10. Apoptosis Measurement

Vip3Aa (31.3, 62.5, and 125 µg/mL) proteins were incubated with *C. albicans* cells for 12 h at 28 °C, and the cells were washed twice with PBS. The cells were stained with PI and FITC-labeled Annexin-V according to manufacturer’s instructions and analyzed using an Attune NxT acoustic focusing cytometer.

### 3.11. Analysis Using SEM

Proteins were incubated with pre-cultivated *C. albicans* at IC_50_ in the presented concentrations and times. Cells were fixed overnight with PBS containing 5% glutaraldehyde at 4 °C. The fixed cells were dehydrated in graded ethanol and critical point-dried under CO_2_. The gold-coated samples were observed using a field emission SEM (JSM-7100F, JEOL Ltd., Tokyo, Japan).

## 4. Conclusions

In summary, we discovered that Vip3Aa has a variety of antifungal effects by pH-, concentration-, and time-dependent structural changes. During the growth of *C. albicans* cells, the intracellular pH varies between 6 and 7, owing to glucose metabolism. The important factor in the antifungal mechanism of Vip3Aa may be its structural variations in the cellular pH. Accordingly, it is difficult to explain the antifungal effect of Vip3Aa with one mechanism. At the initial stage of treatment, it can attach to the cell wall and penetrate the cytosol. The penetrating Vip3Aa can be aggregated in acidic cytosol, suggesting it interferes with the movement of ions and substances into the cell. Morphological defects containing a ring-shape, increased size, and bust may relate to cell wall damaging action. Another mode of antifungal action is proposed, whereby Vip3Aa induces apoptosis of fungal cells via mitochondrial ROS production. This protein may play an important role in the Bacillus defense system against attack by fungal pathogens. Although the MIC value of Vip3Aa is relatively higher than that of other antimicrobial peptides, a synergistic effect will be achieved or antibiotic resistance will be overcome when it is treated in combination with antifungal agents having a different mechanism. However, further research is needed to establish its use in agricultural and clinical applications.

## Figures and Tables

**Table 1:** Antifungal activity of Vip3Aa against fungal cells.

Fungal Strains	Vip3Aa		Melittin	
	µM (µg/mL)			
	IC_50_	MIC	IC_50_	MIC
Yeast				
*C. albicans*	0.7 (62.5)	1.4 (125)	11 (31.3)	22 (62.5)
*C. krusei*	0.7 (62.5)	1.4 (125)	11 (31.3)	22 (62.5)
*C. parapsilosis*	1.4 (125)	2.8 (250)	22 (62.5)	44 (125)
*C. tropicalis *	1.4 (125)	2.8 (250)	22 (62.5)	44 (125)
Molds				
*Colletotrichum gloeosporioides*	5.6 (500)	11.1 (1000)	88 (250)	176 (500)
*F. graminearum*	5.6 (500)	11.1 (1000)	88 (250)	176 (500)
*F. solani*	2.8 (250)	5.6 (500)	88 (250)	88 (250)

## Data Availability

Not applicable.
